# Quick detection of *Carassius auratus* herpesvirus (CaHV) by recombinase-aid amplification lateral flow dipstick (RAA-LFD) method

**DOI:** 10.3389/fcimb.2022.981911

**Published:** 2022-09-12

**Authors:** Lang Gui, Yun Zhao, Dan Xu, Xinyu Li, Jianhua Luo, Wenzong Zhou, Mingyou Li

**Affiliations:** ^1^Key Laboratory of integrated rice-fish farming, Ministry of Agriculture and Rural Affairs, Shanghai Ocean University, Shanghai, China; ^2^Key Laboratory of Exploration and Utilization of Aquatic Genetic Resources, Ministry of Education, Shanghai Ocean University, Shanghai, China; ^3^Eco-environmental Protection Research Institute, Shanghai Academy of Agricultural Sciences, Shanghai, China

**Keywords:** *carassius auratus* herpesvirus (CaHV), tumor necrosis factor receptor (TNFR), recombinase-aid amplification, lateral flow dipstick, point-of-care testing (POCT)

## Abstract

Crucian carp (*Carassius auratus*) is one of the major freshwater species and is also a common food fish in China. Recently, *Carassius auratus* herpesvirus (CaHV) could induce fatal viral disease with high mortality of crucian carp, which had caused huge economic losses. In this study, we described a rapid and simple recombinase-aid amplification (RAA) assay coupled with lateral flow dipstick (LFD), which could achieve sensitive diagnosis of tumor necrosis factor receptor (TNFR) of CaHV within 35 min at 40°C. Our RAA-LFD method had a satisfactory detection limit of 100 gene copies per reaction, which was 100-fold more sensitive than traditional PCR. In addition, no cross-reaction was observed with other viral pathogens, including koi herpesvirus (KHV), cyprinid herpesvirus 2 (CyHV-2), infectious hematopoietic necrosis virus (IHNV), spring viremia of carp virus (SVCV) and grass carp reovirus (GCRV). Furthermore, the overall cost of the method was cut in half compared to previous studies. In conclusion, RAA-LFD assay is therefore, a promising alternative for point-of-care testing (POCT) of CaHV, which is feasible and of certain value in application of aquatic disease control.

## Introduction

Crucian carp (*Carassius auratus*) is a genus of *Carassius* in the family *Cyprinidae* (Xiao et al., 2011). More than 1000 years ago, crucian carp was domesticated in China and introduced into Europe and most parts of the world since the 17th century (Chen, 1956; Balon, 2004; Gao et al., 2012; Podlesnykh et al., 2015). It has the advantages of fast growth, short reproductive cycle, tender meat and high nutritional value (Liao et al., 2013). With the continuous development of breeding technology, the output of crucian carp in China alone has reached 2.7556 million tons in 2019 (Bureau of Fisheries and Fishery Administration under Ministry of Agriculture and Rural Affairs, Nation Fishery Technology Extension Center and C. S. o. Fisheries, 2020).

*Carassius auratus* herpesvirus (CaHV, KU199244) is assigned to family *Alloherpesviridae* and genus *Cyprinivirus*. CaHV is a fatal pathogen of crucian carp, which can cause 100% mortality within one week (Zhang and Gui, 2018; Gui and Zhang, 2019). The isolation of CaHV was firstly reported from the tissues of diseased crucian carp with acute gill hemorrhages in 2016 (Fang et al., 2016). Due to the devastating economic losses, CaHV rapidly becomes a subject for applied research in aquaculture industry. CaHV is a linear double-stranded DNA virus, the entire genome consists of 275,348 bp with 150 predicted open reading frames (ORFs) including tumor necrosis factor receptor (TNFR, ORF 146R) (Zeng et al., 2016).

Aquatic virus including reoviruses, rhabdoviruses and herpesviruses, have brought serious harms to fish and been considered as emerging threats to global aquaculture (Murray, 2012). Cyprinid herpesvirus 2 (CyHV-2) causes acute gill hemorrhage and high mortality in goldfish (*Carassius auratus*) and crucian carp (Tang et al., 2020; Wen et al., 2021). Phylogenetic analysis showed that CaHV was closely related to CyHV-2 with 98.8% similarity (Liu et al., 2018). ORF 146R is a specific gene of CaHV. Koi herpesvirus (KHV) is formally known as cyprinid herpesvirus-3 (CyHV-3), mainly infects common carp (*Cyprinus carpio*) and koi (*Cyprinus carpio koi*) (Hedrick et al., 2005; Yi et al., 2014). Infectious hematopoietic necrosis virus (IHNV) causes clinical disease and mortalities in a wide variety of salmonid species (Dixon et al., 2016). Spring viremia of carp virus (SVCV) has serious effect on crucian carp and grass carp (*Ctenopharyngodon idella*) with 90% mortality (Liu et al., 2022). Grass carp reovirus (GCRV) can cause hemorrhagic disease and result in tremendous loss of grass carp industry (Wang et al., 2012; Wang et al., 2013; Rao and Su, 2015).

Fish herpesviruses diseases have an incubation period, latent or persistent infection is one of the unique characteristics (Zhang and Gui, 2015). Diagnostic assays for CaHV detection were reported, such as multiplex polymerase chain reaction (PCR), paraffin section assay (Fang et al., 2016) and real-time PCR (Li et al., 2019; Zhao et al., 2020). However, precise instruments and trained technicians are required for the traditional detected methods, which are unachievable in limited-resource settings including aquafarms and aqua stores.

Recombinase-aid amplification (RAA) has been a novel isothermal nucleic acid rapid amplification technology in recent years for aquatic diseases pathogen detection, such as IHNV (Chen et al., 2020), CyHV-2 (Preena et al., 2022), GCRV (Wang et al., 2020). The reaction is typically completed in approximately 30 min at 37–42°C (Bei et al., 2010). Lateral flow chromatography strip (LFD) is suited for the visualization of RAA amplicons, as it facilitates analysis of results with the naked eye (Li et al., 2018), and the results can be observed directly in 5-10 minutes (Urusov et al., 2019). Therefore, RAA-LFD method could be applied for point-of-care testing (POCT) for the early infection prevention and control of CaHV.

Based on our previous study of the extraction of DNA from fish skin mucus within 30 seconds with low cost (USD $0.02) (Gui et al., 2022), we established a rapid, sensitive and cheap method to detect CaHV within 35min from sample collection to result interpretation. Our study could provide a simple, rapid, and low-cost detecting application for POCT in aquaculture industry.

## Materials and methods

### Viruses

CaHV DNA was extracted from the crucian carp infected with CaHV, by using skin mucus swabbing and the disc method as we described before (Gui et al., 2022). Briefly, healthy crucian carp were injected intraperitoneally with diseased fish tissue filtrate (viral suspension) kindly provided by Dr. Qiya Zhang (Institute of Hydrobiology, Chinese Academy of Sciences). Other viruses, including CyHV-2 (NC_019495), KHV (NC_009127), IHNV (NC_001652), SVCV (NC_002803), and GCRV-JX01 (MG189638.1), were previously stored in the laboratory. DNA of CyHV-2 and KHV were extracted with a Viral RNA/DNA Extraction Kit (Code No. 9766, Takara Biotechnology Co., Ltd., China), which was carried out in accordance with the instructions of the kit. RNA of GCRV-JX01, IHNV and SVCV were extracted with TRIzol reagent (Invitrogen, USA), which was performed in accordance with the manufacturer’s instructions. Complementary DNA (cDNA) was reverse transcribed by using PrimeScript reverse transcription system (Code No. 2680A, Takara Biotechnology Co., Ltd., China) in accordance with the product protocol, as described in previous study (Wang et al., 2018; Feng et al., 2022). All samples were stored at − 80°C until use.

### Design of primers

The conserved sequences of TNFR (ORF 146R) (GenBank accession no KU199244.1:268307-269281) were chosen as target regions. RAA nucleic acid amplification technology is different from conventional PCR in primer design, the length should be between 30-35 nucleotides (NT) and ideally generate a 100–300 bp amplicon. Thus, five pairs of candidate primers for RAA assays were designed based on RAA primer design principles, the optimal primers were determined by the RAA agarose gel electrophoresis (RAA-AGE) assay. For visualization of the amplified RAA products by a lateral flow dipstick, a DNA probe was designed based on the suitable target sequence between the optimal upstream and downstream RAA primer. Biotin labels were added at 5′-end of the downstream primer to detect the cellular nucleotide. The probe was labeled with fluorophores (FAM) at 5′-end and a polymerase extension blocking group, C3-spacer at 3′-end. In addition, a tetrahydrofuran residue (THF) was added as an internal basic nucleotide analogue. Conventional PCR primers were also designed targeting the conserved sequences of CaHV-TNFR gene by Primer Premier 6.0 software. All primers and probe (**Table 1**) were synthesized and labeled by Suzhou Azenta Biotech Co., Ltd.

**Table 1:** The sequences of primers and probes designed in this study.

Gene name	Prime name	Sequences (5’-3’)	Product size(bp)	Function
TNFR-ORF146R	RAA-F1	TGTTCGACTCATACCCCTACCCACCAGACTA	139	RAA
	RAA-R1	ATCGAGAGCAACGTCGGTTTCCAACCATTCA		
	RAA-F2	CTGTTCGACTCATACCCCTACCCACCAGACTACC	148	
	RAA-R2	ACTCGCGGATCGAGAGCAACGTCGGTTTCCAACC		
	RAA-F3	CTGTTCGACTCATACCCCTACCCACCAGACTAC	138	
	RAA-R3	CGAGAGCAACGTCGGTTTCCAACCATTCACTG		
	RAA-F4	CTAATCGCTGTCCTCTTGACCCTATCGGGCCTGGA	98	
	RAA-R4	GTTGGCTTGGTGATTGCACGTCTGGTGGGTAGTCT		
	RAA-F5	TGCTTCTAATCGCTGTCCTCTTGACCCTATCG	108	
	RAA-R5	TGATTGTTGGCTTGGTGATTGCACGTCTGG		
	RAA-LF	CTGTTCGACTCATACCCCTACCCACCAGACTACC		RAA-LFD
	RAA-LR	Biotin-ACTCGCGGATCGAGAGCAACGTCGGTTTCCAACC		
	RAA-Probe	FAM-CTACCCACCAGACTACCCACCAGACGTGCAA(THF)TCACCAAGCCAACAATC-Spacer		
	PCR-F	ACCCCTACCCACCAGACTAC	789	PCR
	PCR-R	TCGAGGTTCGTTTTGGCGTA		

### Polymerase Chain Reaction (PCR) and construction of recombinant plasmid

Conventional PCR was carried out using PCR-F/R primers specific for TNFR gene of CaHV with product sizes of 789 bp (in Table.1). The cDNA of IHNV, SVCV, GCRV and DNA of CyHV-2, KHV were used as templates to verify the specificity of the established PCR method for CaHV detection, respectively. A 25 µL volume of PCR amplification reaction consisted of 12.5 µL of loading dye mix (Code No. RR003Q, Takara Biotechnology Co., Ltd., China), 1 µL of the primers (10 µmol/L) and 2 µL of DNA template, 8.5 µL ddH_2_O was added at last. The reaction conditions consisted of an initial denaturation at 95 °C/5 min, followed by 32 cycles of denaturation at 95 °C/30s, annealing at 58 °C/30s, extension at 72 °C/50s with a final extension of 72 °C/10 min. Amplicons were visualized by using 1.2% agarose gel electrophoresis, and 100-2000bp DNA marker (Code No. B500350 Sangon Biotech Co., Ltd., Shanghai) was used in experiment.

The TNFR amplicon was inserted into pGEM-T-Easy vector (Promega, Madison, WI, USA), and stored at − 20°C until further use. After identified by sequencing, the concentration of the recombinant plasmid pGEM-T-TNFR was about 80ng/μL by using Nanodrop 2000 (Thermo Fisher Scientific, Waltham, MA, United States). Based on the size of vector (3015 bp) and the insert (789 bp), the copy number was 10^10^ copies/μL converted according to the formula:

Plasmid copy number (copies/μL) = [plasmid concentration(ng/μL) × 10^-9^× (6.02×10^23^)]/[total fragment length(bp)×660g/mol]

Total fragment length = vector length(bp)+fragment length(bp)

### Recombinase aid amplification (RAA) and lateral flow dipstick (LFD) assay

On the basis of the RAA nucleic acid amplification kit (fluorescence method) instructions (ZC Bio-Sci&Tech Co., Ltd., Hangzhou), the RAA reaction system comprised of A buffer 41.5μL, forward primer (10μmol/L) 2μL, reverse primer (10μmol/L) 2μL, template 2μL. These components were added into the lyophilized RAA reaction tube. Then 2.5µL of B buffer (magnesium acetate, 280 mM) was placed on the cap of the reaction tube to commence the reaction. The tube was covered and mixed thoroughly, and centrifuged at low speed for 10 seconds. After reaction at 39°C for 30min, 50μL phenol chloroform was added to purify the amplicons. The purified products were mixed with 6×DNA loading buffer (Sangon Biotech Co., Ltd., Shanghai), and then visualized by 2% agarose gel electrophoresis (AGE).

Additional, LFD (USTAR Biotechnologies Co., Ltd., Hangzhou) and RAA-nfo Kit (ZC Bio-Sci&Tech Co., Ltd., Hangzhou) were utilized for the RAA-LFD assay. 50μL amplification system of RAA was performed according to the manufacturer’s instruction as follows, A buffer 40.9μL, forward primer 2μL, reverse primer 2μL, probe 0.6μL and 2μL template DNA were added into the lyophilized RAA reaction pellets containing the enzyme nfo (Endonuclease IV). Then 2.5µL of B buffer (magnesium acetate, 280 mM) was placed on the cap of the reaction tube to commence the reaction. After mixed properly by centrifugation, the reaction tubes were immediately incubated at 39°C for 30min. LFD assay was used to detect the product of RAA-nfo. 10μL amplified product was mixed with 100ul PBS buffer, and the lateral flow dipstick was inserted into the solution at room temperature. The results could be observed after 5 min. The criteria for optimal reaction conditions were as follows, clear and obvious test line (T) and quality control line (C) formed in LFD strips, indicating that nucleic acid fragments to be detected were contained in the sample. Meanwhile, only quality control line could be recorded in the sample of negative control (Urusov et al., 2019). Therefore, the optimization conditions of RAA-LFD were determined.

### Optimization of RAA-LFD detection method conditions

The recombinant plasmid pGEM-T-TNFR (10^5^ copies/μL) was used as template. The suitable primers were selected after RAA-AGE method. The optimal primer was used for RAA-LFD diagnostic methods. Primer concentration, reaction temperature and incubation time were optimized in subsequent tests. The concentration of primers (RAA-LF/LR) were diluted from 10μM to 0.156μM by two-fold serial dilution, incubated at 39°C for 30min. Then, RAA reactions were performed respectively at 35°C, 36°C, 37°C, 38°C, 39°C, 40°C and 41°C, and the reaction was carried out at the optimal primer concentration for 30 min. Finally, optimal reaction time was also determined, RAA reaction mixtures were incubated respectively in a constant temperature metal bath for 20min, 25min, 30min and 35min at the optimal primer concentration and temperature.

### Sensitivity of RAA-LFD assay

To test the sensitivity of RAA-LFD assay, different copies (10^7^ ~ 10^0^ copies/µL) of the recombinant plasmid were used as templates for both RAA-LFD detection and conventional PCR-AGE detection.

### Specificity of RAA-LFD assay

RAA-LFD assay was performed to detect CaHV and the other fish viruses including CyHV-2, KHV, IHNV, SVCV and GCRV under the optimized conditions.

## Results

### Clone of CaHV TNFR by PCR

**Figure 1:**
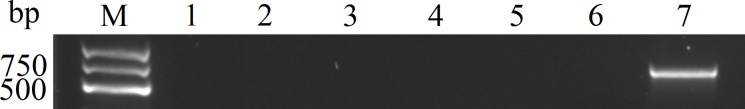
PCR results of different virus with TNFR primer (PCR-F/R). 1, SVCV; 2, GCRV; 3, IHNV; 4, KHV; 5, CyHV-2; 6, negative control (water); 7, CaHV. M: DNA Marker (100~2000 bp).

Only the sample of CaHV generated 789 bp product with successful PCR amplification, which confirmed the specificity of primer pairs (PCR-F/R) to identify CaHV (**Figure 1**). The TNFR amplicon was inserted into pGEM-T-Easy vector and the recombinant plasmid pGEM-T-TNFR was constructed.

### CaHV RAA primer screening by RAA-AGE

**Figure 2:**
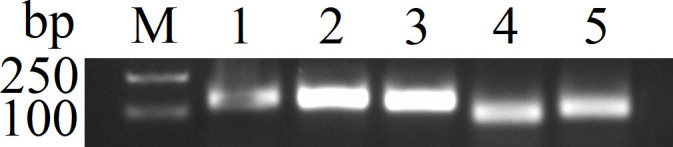
RAA primer screening. 1, RAA-F1/R1 (139bp); 2, RAA-F2/R2 (148bp); 3, RAA-F3/R3 (138bp); 4, RAA-F4/R4 (98bp); 5. RAA-F5/R5 (108bp). M: DNA Marker (100~2000 bp).

Primers RAA-F2/R2 and F3/R3 with product sizes of 148 bp and 138 bp, respectively, gave the highest band intensity (**Figure 2**). However, primer RAA-F2/R2 yielded better results by comparing to primer RAA-F3/R3 during RAA-LFD detection (data not shown), resulted in the selection of RAA-F2/R2 in the remaining experiment. The 148 bp product obtained by RAA-F2/R2 showed 100% similarity to CaHV TNFR (ORF146R) gene by sequencing.

### Optimal reaction conditions of RAA-LFD

**Figure 3:**
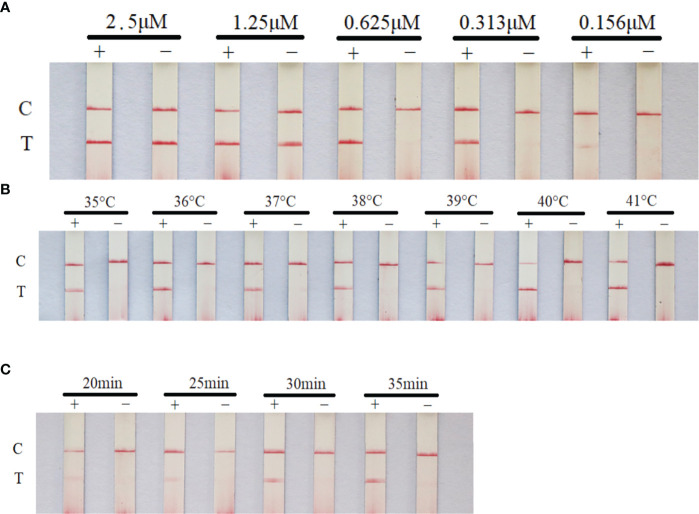
Optimization of RAA-LFD reaction conditions. The optimal primer concentration was 0.625μM, the reaction temperature was 40°C and the reaction time was 30 min. **(A)** screening of primer and probe concentration; **(B)** screening of reaction temperature; **(C)** screening of reaction time. +: positive group (plasmid pGEM-T-TNFR, 10^5^copies/μL). −: negative control (water). C, control line; T, test line.

The primer concentration, reaction time and temperature were optimized in this experiment. The optimal reaction conditions were as follows: quality control line and test line were clear and the brightest, primer concentration was the lowest, and reaction time was the shortest. Thus, the optimal reaction conditions were determined. The primer concentration was 0.625μM, the reaction temperature was 40°C and the reaction time was 30 min (**Figure 3**).

### Sensitivity test

**Figure 4:**
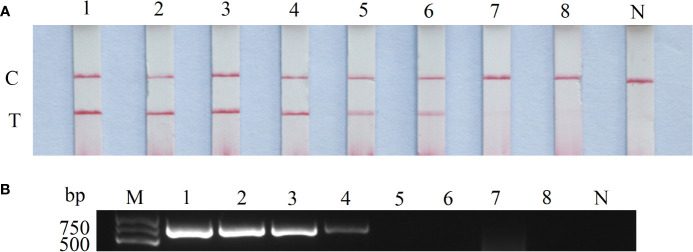
Sensitivity of RAA-LFD and PCR-AGE assays. **(A)** RAA-LFD; **(B)** PCR-AGE; 1-8: 10^7^copies/μL~10^0^ copies/μL. N, Negative control; C, control line; T, test line; M, DNA Marker (100~2000 bp).

The CaHV-harboring plasmids (pGEM-T-TNFR) were 10-fold diluted, and the sensitivity of RAA-LFD and conventional PCR-AGE was compared based on the concentration from 10^7^ to 10^0^ copies/μL. The results shown that the lowest detectable limit was 10^2^ copies/μL for RAA-LFD (**Figure 4A**), and 10^4^ copies/μL for conventional PCR (**Figure 4B**). The sensitivity of RAA-LFD assay was 100 times higher than that of conventional PCR-AGE assay.

### Specificity test

**Figure 5:**
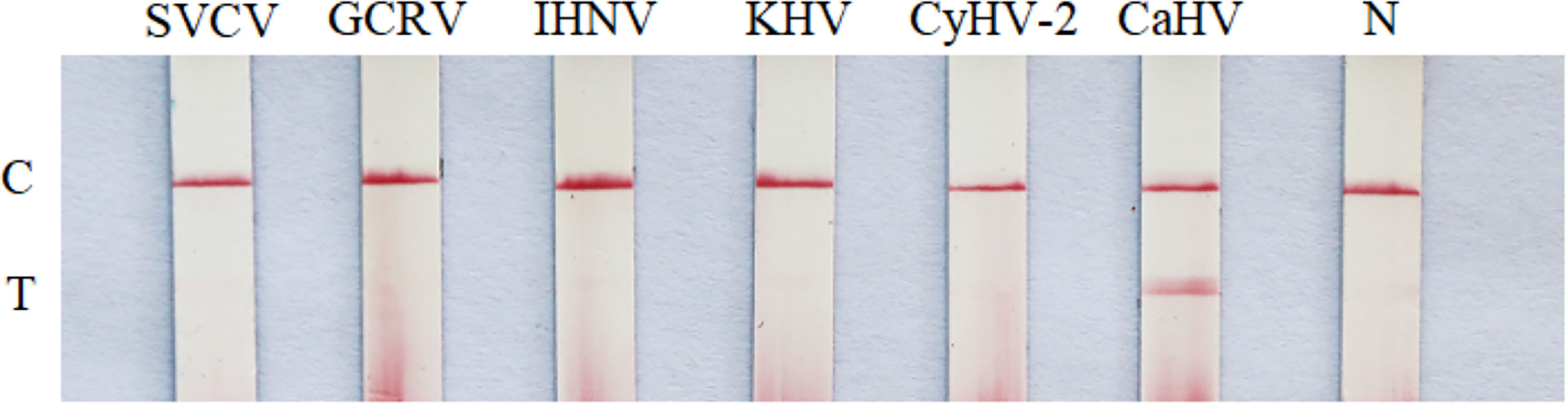
Specificity test of RAA-LFD. N, negative control; C, control line; T, test line.

With the DNA of CaHV, CyHV-2, KHV and cDNA of GCRV, IHNV, SVCV as templates, the products were amplified by RAA-LFD assay. As shown in **Figure 5**, only the RAA product of CaHV showed both test line and quality control line on dipstick. However, the products of other viruses and negative control could only show quality control line, which indicated that the specificity of RAA-LFD assay was good, and this method was tenable.

## Discussion

Fish herpesviruses could cause mild disease in natural conditions, which are responsible for severe losses of aquaculture industry in recent years (Gotesman et al., 2013; Xu et al., 2013). Over 14 herpesviruses have been considered to be associated with disease outbreaks in fish recent years (Kibenge, 2019). Therefore, it is very important to explore a specific, sensitive and cheap method to better prevent and control fish herpesviruses

Several specific and sensitive methods have been developed for detection of viruses in fish, such as reverse transcription (RT)-PCR, nested PCR test, real-time PCR (qPCR), reverse-transcriptase real-time PCR (RT-rPCR) and reverse-transcriptase droplet digital PCR (RT-ddPCR) (Gilad et al., 2004; El-Matbouli et al., 2007; Sadler et al., 2008; Honjo et al., 2010; Yuasa et al., 2012; Purcell et al., 2013; Zeng et al., 2014; Jia et al., 2017). However, the requirement of specialized equipment and trained technicians of those methods limit the applications outside the laboratory environment.

Isothermal amplification technologies, such as RPA/RAA and loop mediated isothermal amplification (LAMP) assays, have features of high specificity, rapidity and simplicity of detection of various viruses (Congdon et al., 2019; Fan et al., 2020). In comparison between RPA/RAA and LAMP, RPA just demands a single pair of primers and lasts around 30 min, whereas LAMP requires 6 primers and lasts around 60 min, which illustrates that RPA is more convenient and economical than LAMP for aquatic diseases detection (Zhang et al., 2014; Lobato and O’Sullivan, 2018; Diagne et al., 2020). Therefore, the feasibility of RPA/RAA assay for CaHV detection was assessed, and the obtained results were compared with those of PCR as the reference method.

Among the various visualization methods for RAA/RPA amplicons, LFD is an endpoint detection technology for visual observation of amplification products for POCT. We optimized the reaction conditions of RAA-LFD assay, the results showed that high concentration of primers would cause false positive results (**Figure 3A**), which might due to the false-positive signals from primer–dimers (Yang et al., 2020). RAA-LFD assays demonstrated excellent sensitivity by facilitating the detection of low copy numbers of the CaHV-harboring plasmids (pGEM-T-TNFR). Our RAA-LFD assay showed a comparable limit of detection (100 copies), exhibited by most of the RPA assays developed for fish viruses, such as largemouth bass ranavirus (58.3 copies) (Guo et al., 2022), CyHV-2 (100 copies) (Preena et al., 2022) and *Micropterus salmoides* rhabdovirus (170 copies) (Feng et al., 2022). However, it needs to be further verified by testing more samples of natural infection before being practically applied as routine diagnostic methods.

RPA-LFD assays are relatively expensive by comparing to conventional PCR methods, which would last more than 1h and cost approximately $20 each reaction from DNA sample extraction to visualized result obtaining (Qin et al., 2021). However, based on a swabbing and disc method for DNA extraction established in our lab (Yi et al., 2014), the total reaction time from DNA extraction to RAA-LFD assay could be completed within 35 min and cost lower than USD $10. Therefore, our method has a great potential to be as a useful tool for reliable and quick POCT of CaHV infection, especially in resource-limited conditions such as aqua farms and stores.

## Conclusions

A simple and reliable RAA-LFD assay detecting CaHV was developed for the first time which could accomplish successful detection of 100 copies of the viral TNFR gene within 35 min at 40°C. This presents a rapid and sensitive POCT of CaHV under resource-limited conditions.

## Data availability statement

The datasets presented in this study can be found in online repositories. The names of the repository/repositories and accession number(s) can be found in the article/supplementary material.

## Ethics statement

All handling of fish in this study was conducted in accordance with the guidelines of the Shanghai Ocean University Animal Care and Use Committee with approval number SHOU-2021-118.

## Author contributions

Conceptualization, LG and ML. Methodology, YZ and XL. Software, YZ. Validation, DX. Formal analysis, YZ and JL. Investigation, LG. Resources, WZ, LG, and ML. Data curation, YZ. Writing—original draft preparation, LG and YZ. Writing—review and editing, ML and LG. Visualization, DX. Supervision, ML. Project administration, YZ. Funding acquisition, WZ. All authors contributed to the article and approved the submitted version.

## Funding

LG provided the funding of the National Key Research and Development Program of China (2018YFD0900601 and 2018YFD0900302) and the Science and Technology Commission of Shanghai Municipality (21ZR1427200), both of which were received from government. The funder designed the experiment and reviewed the manuscript. WZ provided the funding of the China Agriculture Research System of MOF and MARA (CARS-46) which was received from government. The funder did the analysis.

## Conflict of interest

The authors declare that the research was conducted in the absence of any commercial or financial relationships that could be construed as a potential conflict of interest.

The reviewer WH declared a shared affiliation, with no collaboration, with several of the authors, LG, YZ, DX, XL, and ML, to the handling editor at the time of review.

## Publisher’s note

All claims expressed in this article are solely those of the authors and do not necessarily represent those of their affiliated organizations, or those of the publisher, the editors and the reviewers. Any product that may be evaluated in this article, or claim that may be made by its manufacturer, is not guaranteed or endorsed by the publisher.
